# Constitutive Type VI Secretion System Expression Gives *Vibrio cholerae* Intra- and Interspecific Competitive Advantages

**DOI:** 10.1371/journal.pone.0048320

**Published:** 2012-10-26

**Authors:** Daniel Unterweger, Maya Kitaoka, Sarah T. Miyata, Verena Bachmann, Teresa M. Brooks, Jessica Moloney, Oscar Sosa, David Silva, Jorge Duran-Gonzalez, Daniele Provenzano, Stefan Pukatzki

**Affiliations:** 1 Department of Medical Microbiology and Immunology, University of Alberta, Edmonton, Alberta, Canada; 2 Department of Biomedical Sciences, University of Texas Brownsville, Brownsville, Texas, United States of America; Université de Genève, Switzerland

## Abstract

The type VI secretion system (T6SS) mediates protein translocation across the cell membrane of Gram-negative bacteria, including *Vibrio cholerae* – the causative agent of cholera. All *V. cholerae* strains examined to date harbor gene clusters encoding a T6SS. Structural similarity and sequence homology between components of the T6SS and the T4 bacteriophage cell-puncturing device suggest that the T6SS functions as a contractile molecular syringe to inject effector molecules into prokaryotic and eukaryotic target cells. Regulation of the T6SS is critical. A subset of *V. cholerae* strains, including the clinical O37 serogroup strain V52, express T6SS constitutively. In contrast, pandemic strains impose tight control that can be genetically disrupted: mutations in the quorum sensing gene *luxO* and the newly described regulator gene *tsrA* lead to constitutive T6SS expression in the El Tor strain C6706. In this report, we examined environmental *V. cholerae* isolates from the Rio Grande with regard to T6SS regulation. Rough *V. cholerae* lacking O-antigen carried a nonsense mutation in the gene encoding the global T6SS regulator VasH and did not display virulent behavior towards *Escherichia coli* and other environmental bacteria. In contrast, smooth *V. cholerae* strains engaged constitutively in type VI-mediated secretion and displayed virulence towards prokaryotes (*E. coli* and other environmental bacteria) and a eukaryote (the social amoeba *Dictyostelium discoideum*). Furthermore, smooth *V. cholerae* strains were able to outcompete each other in a T6SS-dependent manner. The work presented here suggests that constitutive T6SS expression provides *V. cholerae* with an advantage in intraspecific and interspecific competition.

## Introduction

The Gram-negative bacterium *Vibrio cholerae* is the causative agent of the acute diarrheal disease cholera and remains a serious health risk to humans. In addition to the two main virulence factors needed to cause massive watery diarrhea–cholera toxin [1] and the toxin coregulated pilus [2] –the bacterium utilizes accessory virulence factors also capable of causing diarrheal disease. Accessory toxins such as hemolysin (HlyA) and actin-cross-linking repeats-in-toxin (RtxA) have been reported to be virulence mechanisms exploited by some strains [3].

Another such accessory virulence factor is the type VI secretion system (T6SS), which confers cytotoxic effects against both prokaryotic and eukaryotic cells [4]–[6]. Bacteria have developed numerous mechanisms to export proteins, including toxins, across their cell walls into the surrounding environment or into host cells. To date, six distinctive pathways, collectively called secretion systems and classified into type I to type VI (T1SS – T6SS), have been identified in Gram-negative bacteria [7]. The T6SS of *V. cholerae* mediates cytotoxicity towards eukaryotic hosts, including murine macrophages [5], [8], [9] and the amoeba *Dictyostelium discoideum* [4]. The *V. cholerae* T6SS is encoded by three gene clusters on two separate chromosomes: one large cluster (VCA0107 – VCA0124) [10] and two small auxiliary clusters (VCA0017 – VCA0021 and VC1415 – VC1421). Bioinformatic analyses and a series of experimental approaches have elucidated the functions of several genes belonging to the *V. cholerae* T6SS clusters. For example, the Hcp protein [11], secreted by bacteria with a functional T6SS, forms a nanotube structure with an internal diameter of 4 nm [12]. Three VgrG proteins were shown to interact with each other to form a trimeric complex that structurally resembles a T4-bacteriophage gp5-gp27 tail spike complex [9], but unlike their phage counterparts lack an internal channel [13]. The current working model of the T6SS is based on these observations and the finding that Hcp and VgrG are codependent for secretion. The model proposes that the Hcp nanotube, decorated with a VgrG trimer at its top, is pushed through the bacterial envelope of the predator cell and into the prokaryotic or eukaryotic target cell. It is suggested that cytoplasmic VipA and VipB (VCA0107 and VCA0108) form a contractile sheath around the Hcp tube similar to the T4 phage outer sheath; contraction of the VipAB sheath ejects the Hcp tube from the predator cell [14]. The VgrG cap might mediate toxicity via the C-terminal extensions of evolved VgrGs upon delivery into the target cell [5]. Alternatively, the cap might dissociate from the Hcp nanotube to allow delivery of soluble toxin(s) or effector molecule(s) through the Hcp conduit [13]. VasH (VCA0117) acts as a sigma-54 activator protein and controls transcription of T6SS genes including *hcp* and *vgrG*. We recently reported that the *V. cholerae* T6SS also exerts contact-dependent killing properties against other Gram-negative bacteria such as *Escherichia coli* [6]. This finding suggests that *V. cholerae* may employ the T6SS to compete with commensal bacteria in the human intestine and/or environmental reservoirs.

**Table 1:** Bacterial strains and plasmids.

Strain or plasmid	Description	Reference or source
Strains		
* Vibrio cholerae* V52	O37 serogroup strain, *ΔhapA, ΔrtxA, ΔhlyA*, smR	[25]
* Vibrio cholerae* V52*ΔvasK*	V52 mutant lacking *vasK* (VCA0120)	[25]
* DL2111, DL2112*, *DL4211, DL4215*	Environmental isolates collected in this study (see Table 3).	This study
* DL4211* Δ*vasK*	DL4211 mutant lacking *vasK* (VCA0120)	This study
* DL4215* Δ*vasK*	DL4215 mutant lacking *vasK* (VCA0120)	This study
* Escherichia coli* DH5α λpir	*fhuA2* Δ*(argF-lacZ)U169 phoA glnV44 Φ80* Δ*(lacZ)M15 gyrA96 recA1 relA1 endA1 thi-1 hsdR17*	Provenzano Laboratory (University of Texas at Brownsville)
* Escherichia coli* SM10λpir	KmR, thi-1, thr, leu, tonA, lacY, supE, recA::RP4-2-Tc::Mu, pir	Mekalanos Laboratory (Harvard Medical School)
* Escherichia coli* MG1655	F- lambda- *ilvG*- *rfb*-50 *rph*-1, *Rif^R^*	Raivio Laboratory (University of Alberta)
* Klebsiella pneumoniae*	Wild-type. T6SS-negative control	Kessin Laboratory (Columbia University)
Plasmids		
pBAD18 pBAD18-*vasH::myc*	pBAD vector, pBR322 ori, *araC*, Kan^R^ pBAD18 carrying *vasH* (VCA0117) of the *Vibrio cholerae* strain V52	[39] [16]
pBAD24 pBAD24-vasK	pBAD vector, pBR322 ori, *araC*, Amp^R^ pBAD24 carrying *vasK* (VCA0120) of the *Vibrio cholerae* strain V52	[39] [6]
pWM91	*oriR6K mobRP4 lacI* p*tac tnp* mini-Tn*10*Km; Km^r^ Amp^r^	[23]
pGEM-T-easy	Vector for cloning PCR products, Amp^R^	Promega

The environmental reservoirs of *V. cholerae* (river deltas with brackish waters, oceans, and deep seas [15]) are as diverse as the genomic content of this bacterium. The *V. cholerae* pangenome is estimated to consist of ∼6,500 genes [16]. Because all *V. cholerae* genomes sequenced so far contain the three gene clusters encoding the T6SS, we conclude that the T6SS belongs to the 1,500-gene core genome. Although the T6SS appears to be conserved in *V. cholerae*, the system is regulated differently between strains. While the O37 serotype V52 strain expresses T6SS genes constitutively, the O1 El Tor strain C6706 represses its T6SS under laboratory conditions. Mutations in the genes encoding the transcriptional regulator TsrA (VC0070) and the quorum sensing system regulator LuxO (VC1021) are required for T6SS expression in C6706 under laboratory conditions [17]. Another El Tor strain, A1552, activates its T6SS when grown under high osmolarity conditions and/or low temperature [18]. A transcriptional regulator encoded within the T6SS gene–cluster is VasH (VCA0117). As a sigma-54 activator protein, VasH controls the expression of T6SS genes including *hcp* and *vgrG* [19], [20]. These differences in T6SS regulation led us to investigate whether *V. cholerae* strains employ constitutive or restricted T6SS regulation in defined environmental reservoirs. We focused on the Rio Grande, a river that empties into the Gulf of Mexico and is considered to be a major reservoir of unique, nonpandemic O1 El Tor strains responsible for sporadic food-borne cholera in the summer [21]. Environmental *V. cholerae* isolates (RGVCs) collected at two locations along the Rio Grande were examined to test whether constitutive T6SS expression is prevalent in *V. cholerae* exposed to microbial competitors and predators.

**Table 2:** Primers.

PRIMER	OLIGONUCLEOTIDE SEQUENCE (restriction sites underlined)
5′vasH	GAATTC ACCATGAGTCAATGGCTGGCG
3′-vasH-myc	CCTCTAGATCATAAATCTTCTTCAGAAATTAATTTTTGTTCTGGGGTTTTGATCTCCAA
5′-vasK-pBAD24	TTTGAATTCACCATGTGGAAATTCATT
3′-vasK-pBAD24	TTTTCTAGATTAATAGAGTGTTTTAGAC
5′-16S Universal (E8F)	AGAGTTTGATCCTGGCTCAG
3′-16S Universal (U1115R)	AGGGTTGCGCTCGTTG

## Materials and Methods

### Strains and Culture Conditions

A streptomycin-resistant *V. cholerae* strain V52 (O37 serogroup) lacking *hapA*, *rtxA*, and *hlyA* genes [4] was used as a T6SS-positive strain in all experiments presented in this study. DH5αλpir and SM10λpir were used for cloning, and mating of pWM91-based plasmids, respectively. The strains and plasmids used in this study are listed in Table 1. Unless stated otherwise, bacteria were grown in a Luria-Bertani (LB) broth at 37°C with shaking (200 rpm). Rifampicin-resistant (50 µg·mL^−1^) *Vibrio communis, Vibrio harveyi*, and *Pseudoalteromonas phenolica* were grown in ½ YTSS broth (2.5 g·L^−1^ tryptone, 4 g·L^−1^ yeast extract, 20 g·L^−1^ sea salts (Sigma)) at 30°C. Antibiotic concentrations used to maintain the plasmids were 100 µg·mL^−1^ ampicillin or 50 µg·mL^−1^ kanamycin. *D. discoideum* AX3 cells were obtained from the Dicty Stock Center and maintained in liquid culture (HL5) with shaking (150 rpm) at 22°C [22]. Environmental bacteria were collected by submerging a Turtox tow net (Envco, New Zealand) with a 20 µm pore-size Nitex mesh spanning a 30.48 cm diameter mouth in estuary water for one minute. Water samples (200 mL) collected from estuaries of the Rio Grande delta were blended with a handheld homogenizer (PRO Scientific; Oxford, CT), and vacuum filtered through Whatman filter paper number 3 (GE Healthcare, Little Chalfont, UK). A second vacuum filtration was performed on the filtrate through 0.45 µM pore-size membranes (Millipore, Bedford, MA). Filters were incubated separately in a small volume of 0.15 M sterile NaCl for one hour shaking at RT. The suspensions were plated on thiosulfate-citrate-bile salts-sucrose (TCBS) agar (BD, Franklin Lakes, NJ) and/or marine agar 2216 (BD, Franklin Lakes, NJ). Following incubation for 16 hours at 30°C, colony forming units (CFUs) were isolated and cultured in LB broth. A polymorphic 22-kb region was sequenced for both isolates, DL2111 and DL2112, for strain identification. Sequences were submitted to GenBank (accession number JX669612 and JX669613).

**Table 3:** RGVC isolates.

DL Number	Serogroup	VasH sequence compared to V52
2111	None (rough)	frameshift, H116D, Q278L, T449A, T456I
2112	None (rough)	frameshift, H116D, Q278L, T449A, T456I
4211	O123	H116D, T449A
4215	O113	H116D, T441S, P447S, T449V
N16961	O1	H116D, T449A

### DNA Sequence Analysis and Protein Structure Prediction Analysis

Nucleotide sequence analyses and alignments were performed with MacVector software (version 11.0.2).

**Figure 1:**
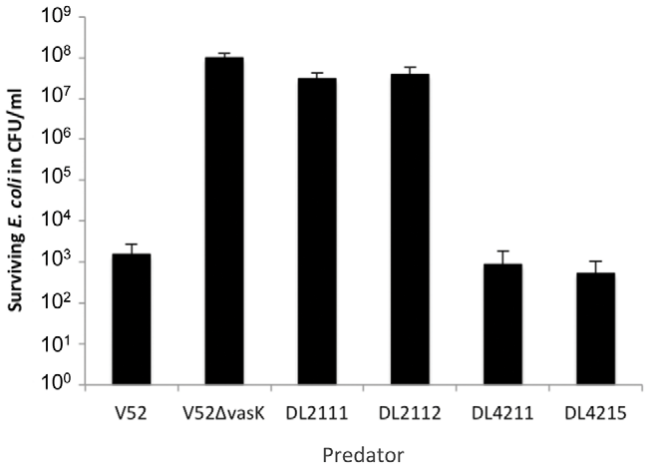
Ability of RGVC isolates to kill *E. coli*. Rough RGVC isolates DL2111 and DL2112, and smooth RGVC isolates DL4211 and DL4215 were tested for their ability to confer T6SS-mediated prokaryotic killing. V52 and V52Δ*vasK* were used as virulent and avirulent controls, respectively. *V. cholerae* and *E. coli* were mixed in a 10∶1 ratio and incubated for 4 hours at 37°C. Bacterial spots were resuspended, serially diluted, and plated on *E. coli*-selective media to determine the number of surviving *E. coli*. The averages and standard deviations of two independent experiments, each performed in duplicates are shown.

### 16S Ribosomal Sequencing

Primers binding to conserved 16S ribosomal gene sequences were used to PCR-amplify the 16S ribosomal sequences from environmental bacterial isolates. Primer sequences are summarized in Table 2. DNA sequencing was performed at the University of Alberta Applied Genomics Centre and species were identified using BLASTn.

**Figure 2:**
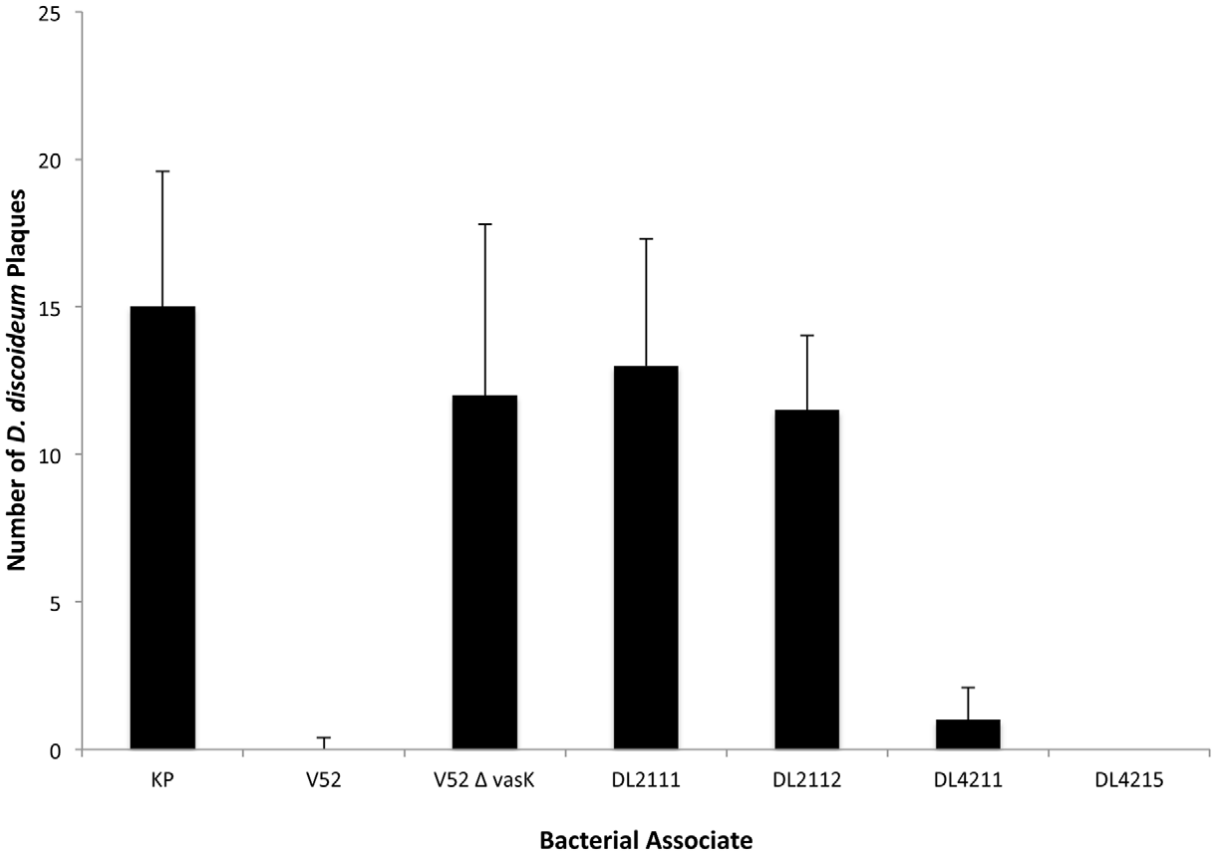
RGVC isolates with a constitutive T6SS kill *D. discoideum*. 10^3^ *D. discoideum* cells were plated with indicated bacteria on SM/5 agar plates that support bacterial but not amoeboid growth. Plaques formed by *D. discoideum* were counted on the third day of incubation. The graph summarizes the results of two independent experiments. Standard deviations are shown. KP: *Klebsiella pneumoniae*.

### Protein Secretion Profiles

Overnight cultures of bacterial strains were diluted to 1∶100 in 3 mL of fresh LB containing appropriate antibiotics and incubated until they reached late mid-logarithmic growth phase (OD_600_ ∼0.6). L-arabinose (0.1%) was added to induce expression of the P_BAD_ promoter in pBAD24 and pBAD18. Bacteria were pelleted at high speed in a tabletop microcentrifuge for 5 minutes. Supernatants were filtered through 0.22 µm low protein-binding polyvinylidine fluoride (PVDF) syringe filters (Millipore). Proteins were precipitated with 20% trichloroacetic acid (TCA) for 15 minutes on ice, pelleted by centrifugation at 14,000× g for 5 minutes at 4°C, and washed twice with ice-cold acetone to remove residual TCA. Protein pellets were resuspended in 40 µL SDS-PAGE lysis buffer (40% glycerol; 0.24 M Tris-HCl, pH 6.8; 8% SDS; 0.04% bromophenol blue; 5% β-mercaptoethanol) and boiled for 10 minutes. 300 µL of bacterial culture was centrifuged at 14,000× g for 5 minutes. Bacterial pellets were resuspended in an equal volume of lysis buffer and boiled for 10 minutes. Samples were subjected to SDS-PAGE (10% acrylamide) and analyzed by western blotting using a rabbit polyclonal antibody against DnaK (Stressgen, diluted 1∶15,000), mouse anti-RNAP (Neoclone, diluted1∶1000), mouse anti-beta-lactamase (Sigma, diluted 1∶200), and polyclonal rabbit anti-Hcp [5] antiserum (diluted 1∶500). Secondary antibodies used were goat anti-mouse horseradish peroxidase (HRP) and goat anti-rabbit HRP (both Santa Cruz, diluted 1∶3000).

**Figure 3:**
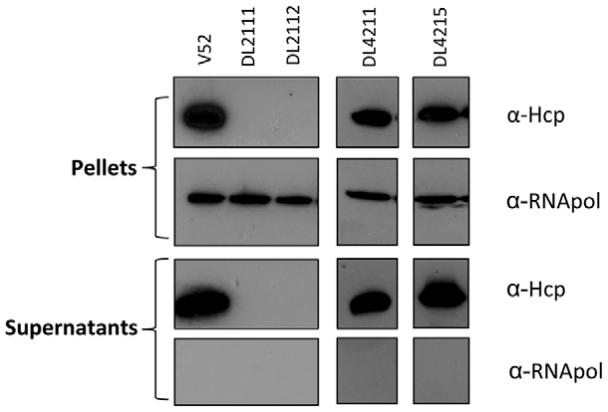
RGVC isolates differ in T6SS regulation. Indicated RGVC isolates and V52 (positive control) were cultured to midlogarithmic phase of growth followed by centrifugal separation of pellets and culture supernatants. Supernatant portions were concentrated by TCA precipitation and both fractions were subjected to SDS-PAGE followed by western blotting using the antibodies indicated. Experiments were repeated at least three times with equivalent results.

### *D. discoideum* Plaque Assays

100 µL of overnight bacterial culture and 10^3^ *D. discoideum* AX3 cells were spread on SM/5 plates [22]. Arabinose (0.1%) was added to SM/5 plates when indicated. Plates were incubated at 22°C for 3 days to assess the number of plaques.

**Figure 4:**
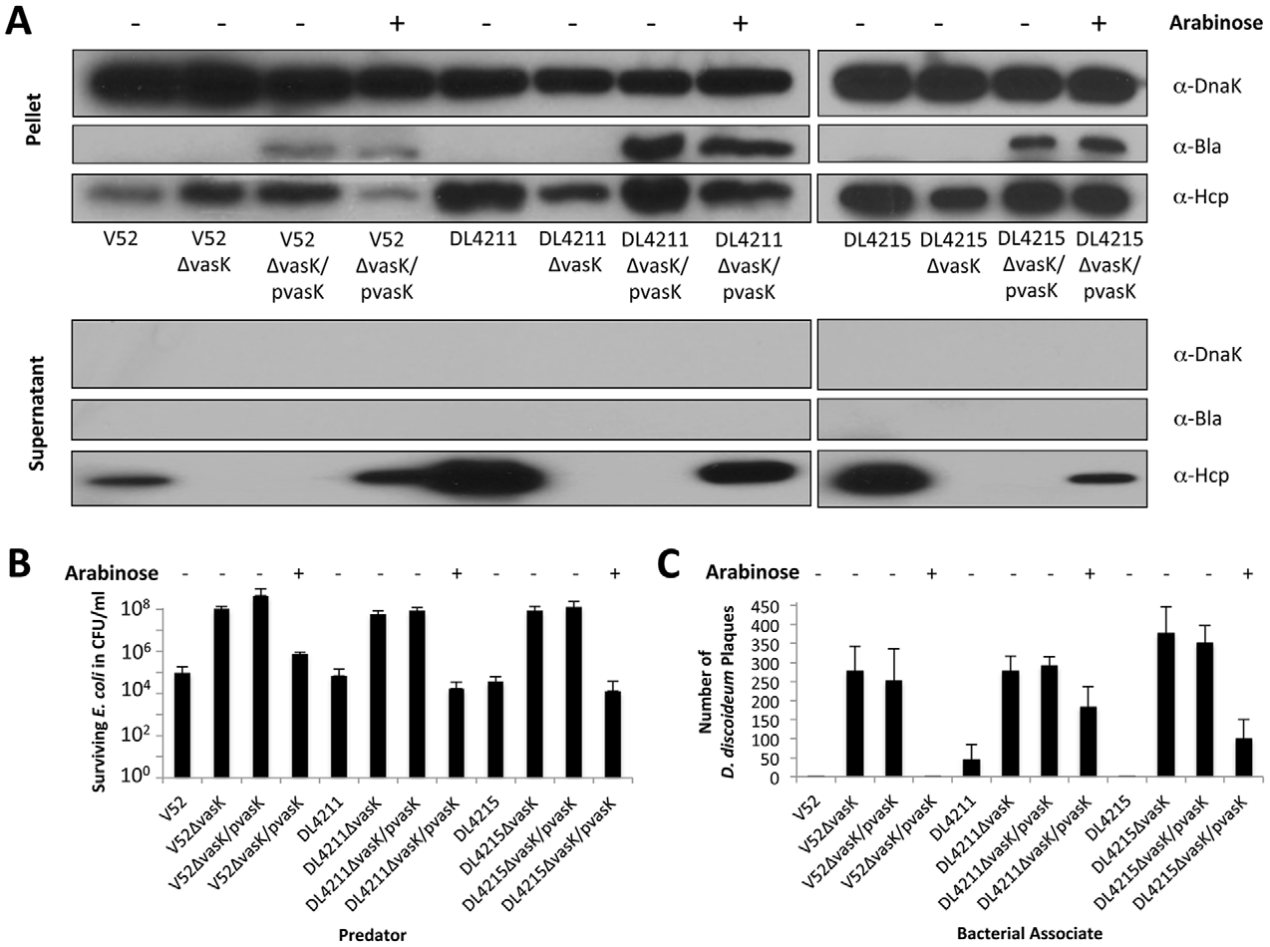
Complementation of a *vasK* null-mutation restores T6SS-dependent secretion and virulence. (A) *VasK*-mutants of smooth RGVC isolates carrying a plasmid for arabinose-induced *vasK* expression were cultured to midlogarithmic phase of growth in the presence or absence of 0.1% arabinose. V52 and the isogenic *vasK* mutant were used as positive and negative controls, respectively. Pellets and culture supernatants were separated by centrifugation. The supernatant portions were concentrated by TCA precipitation and both fractions were subjected to SDS-PAGE followed by western blotting using the antibodies indicated. (B) Survival of *E. coli* MG1655 after mixing with *V. cholerae*. *V. cholerae* and *E. coli* were mixed in a 10∶1 ratio and incubated for 4 hours at 37°C before the resulting spots were resuspended, serially diluted, and plated on *E. coli*-selective media. Data represent the averages of three independent experiments. Standard deviations are included. (C) Survival of *D. discoideum* after mixing with *V. cholerae*. *D. discoideum* was plated with *V. cholerae* and the number of plaques formed by surviving *D. discoideum* were counted after a 3-day incubation at 22°C. Data are representative of three independent experiments. Standard deviations are shown.

### Bacterial Killing Assay

Bacterial strains were grown as lawns on LB-agar plates with appropriate antibiotics. Environmental non-*V. cholerae* strains were grown on 1/2 YTSS agar plates with appropriate antibiotics. Streptomycin-resistant (rifampicin-sensitive) predator and rifampicin-resistant (streptomycin-sensitive) prey were harvested and mixed at a 10∶1 ratio with volumes normalized by OD_600_ readings. 25 µL of the mixed bacterial culture was spotted onto prewarmed LB-agar (or 1/2 YTSS agar plates for mixtures containing non-*V. cholerae* strains) and incubated at 37°C (or 30°C for non-*V. cholerae* strains) for 4 h. Bacterial spots were harvested and the CFU·mL^−1^ of surviving prey and predator were measured by serial dilution and selective growth on agar containing 50 µg·mL^−1^ rifampicin and 100 µg·mL^−1^ streptomycin, respectively. Where applicable, arabinose was added to LB plates at a final concentration of 0.1% to induce expression from the P_BAD_ promoter during the 4 hour incubation.

**Figure 5:**
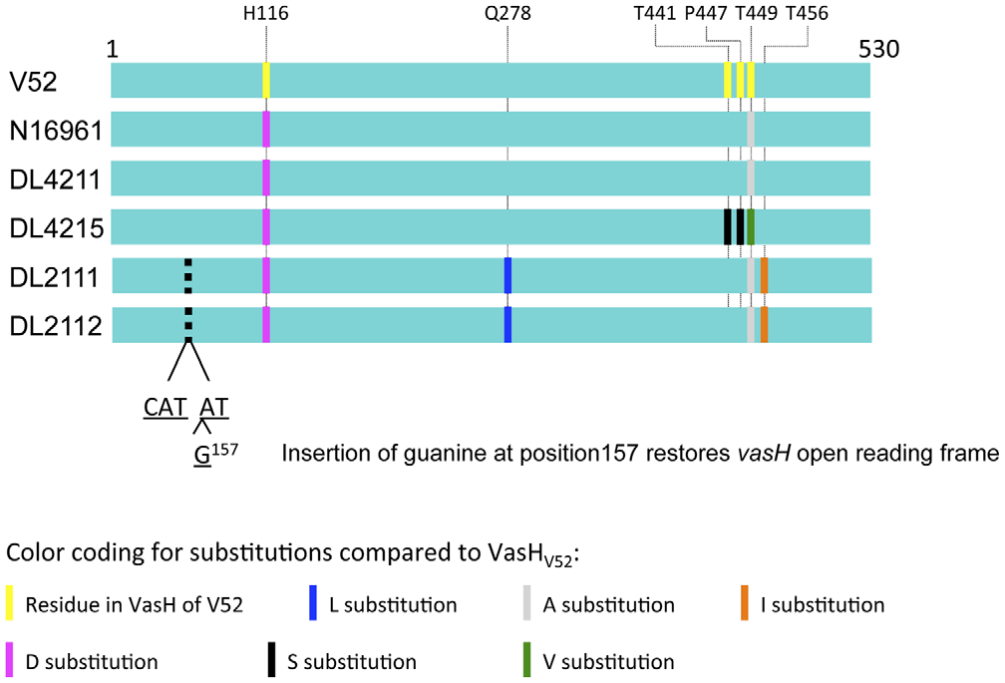
Alignment of VasH polypeptide sequences of RGVC isolates. VasH of V52, N16961, and four RGVC isolates were aligned. In the rough isolates, a guanine was inserted at position 157 of *vasH* to restore the open reading frame. Colored bars indicate substitutions compared to VasH from V52.

### DNA manipulations

3′-Myc-tagged *vasH* was PCR-amplified from *V. cholerae* V52 chromosomal DNA with primers 5′vasH and 3′vasH::myc (Table 1). The resulting PCR product was restricted with 5′-*Eco*RI and 3′-*Xba*I, cloned into pGEM T-easy (Promega), and subcloned into pBAD18.

**Figure 6:**
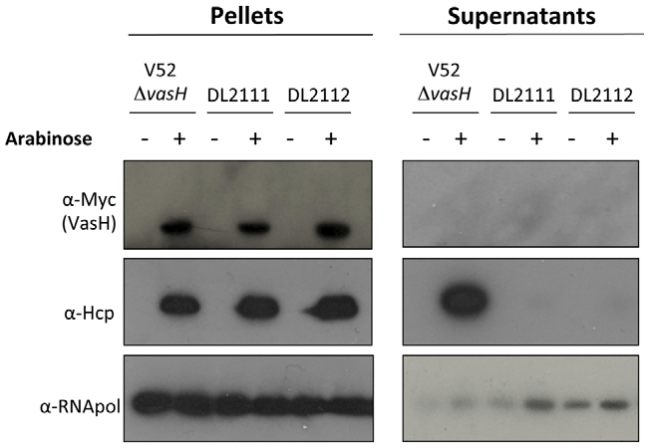
*VasH* complementation restores Hcp synthesis but not secretion in rough RGVC isolates. *V. cholerae* isolates were transformed with pBAD18-vasH::myc. The isolates were cultured to midlogarithmic phase of growth in the presence or absence of 0.1% arabinose. Pellets and culture supernatants were separated by centrifugation. The supernatant portions were concentrated by TCA precipitation and both fractions were subjected to SDS-PAGE followed by western blotting using the antibodies indicated. Data are representative of three independent experiments.

In-frame deletion of *vasK* was performed as described by Metcalf et al. [23] using the pWM91-based *vasK* knockout construct [9]. During sucrose selection, sucrose concentration was increased from 6% to 20% for all RGVC gene deletions because these isolates exhibited increased tolerance to sucrose compared to V52.

**Figure 7:**
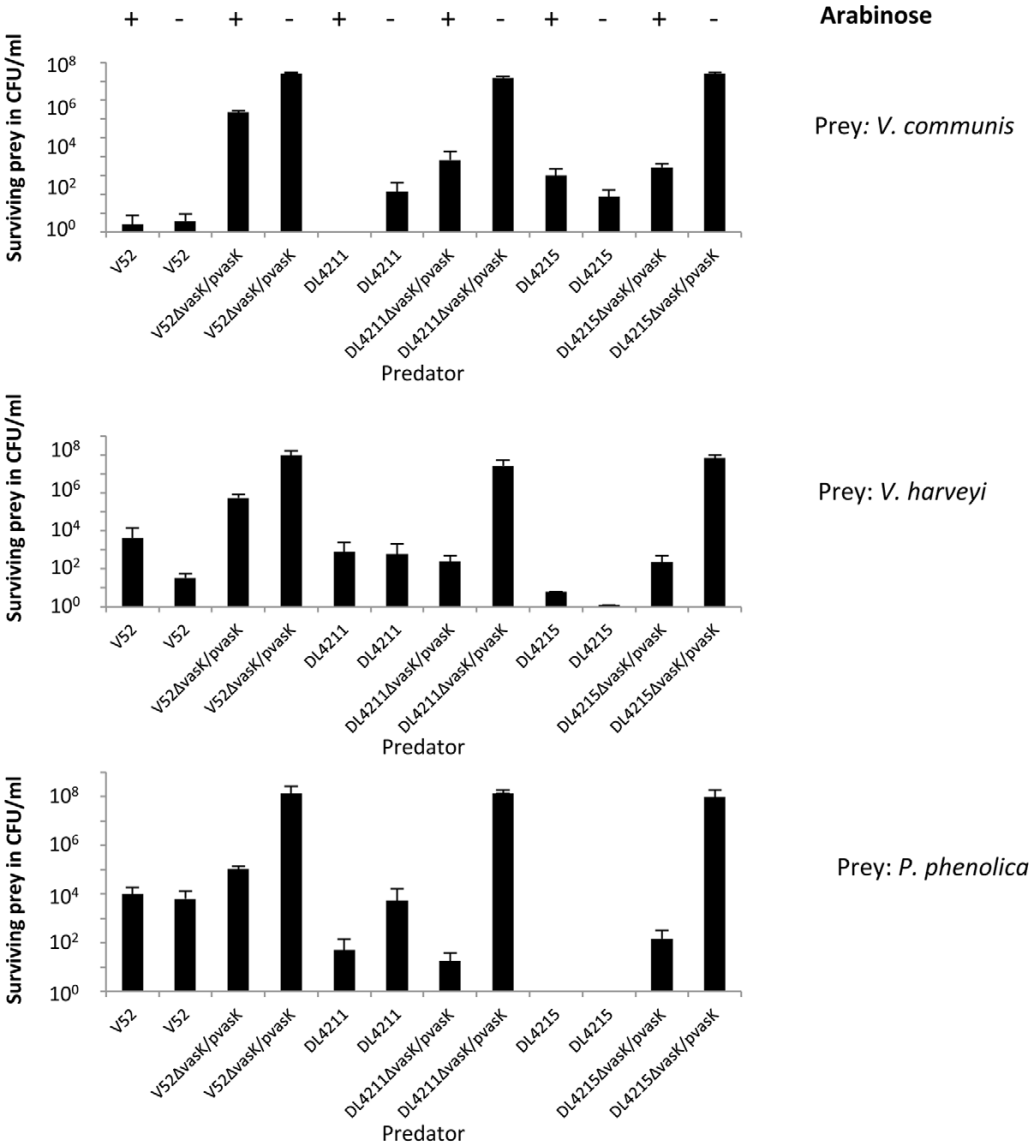
RGVC isolates kill bacterial neighbors. *V. cholerae* and prey bacteria were mixed in a 10∶1 ratio and incubated on ½ YTSS agar for 4 hours at 30°C. Bacterial spots were resuspended, serially diluted, and plated on selective YTSS agar to determine the number of surviving prey. The average and standard deviations of three independent experiments, each performed in duplicates, are shown.

For complementation, *vasK* was amplified from V52 chromosomal DNA using primers 5′-vasK-pBAD24 and 3′-vasK-pBAD24 (Table 1). The resulting PCR product was purified using the Qiagen PCR cleanup kit, digested with *Eco*RI and *Xba*I, and cloned into pBAD24.

**Figure 8:**
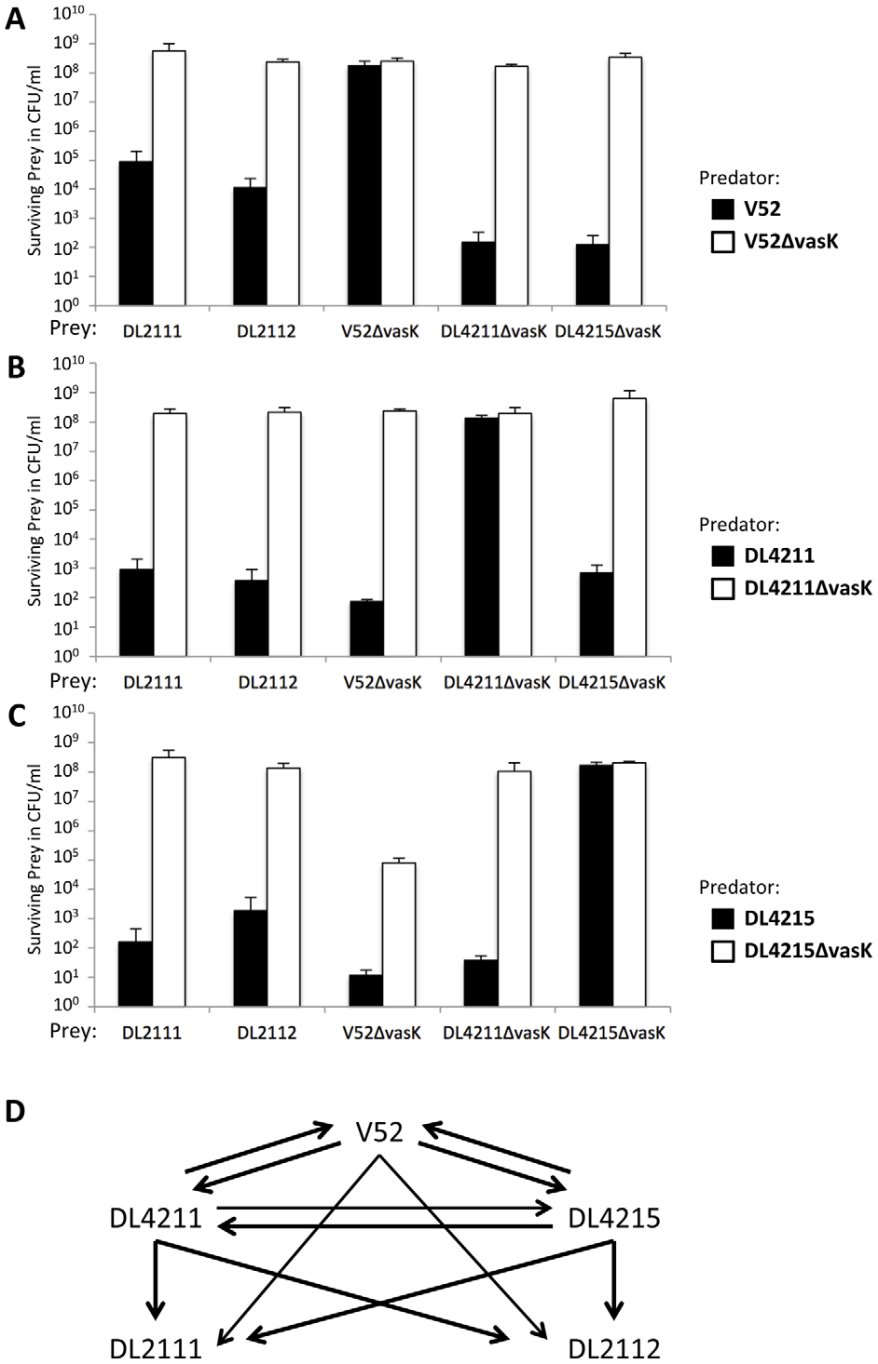
T6SS-dependent competition among *V. cholerae* isolates. (A–C) Smooth *V. cholerae* isolates successfully competed with each other and outcompeted the rough isolates in a T6SS-dependent manner. All combinations among the isolates and their isogenic *vasK* mutants were tested in a killing assay: Predator- and prey-*V. cholerae* were mixed in a 10∶1 ratio and incubated for 4 hours at 37°C. Bacterial spots were resuspended, serially diluted, and plated on selective media to determine the number of surviving prey. The number of surviving prey in the presence of T6SS^+^ or T6SS^−^ predator are shown. (D) Arrows indicate the competitive relationship between isolates such that the arrow points from the predator towards the prey. Arrow thickness indicates relative killing efficiency. T6SS-dependence of the killing phenotype was confirmed by employing the *vasK*-deficient predator of each *V. cholerae* isolate examined. To avoid killing of the predator, *vasK*-deficient prey of smooth T6SS^+^ isolates were used. The average and standard deviations of two independent experiments, each performed in duplicates, are shown.

**Table 4:** Secretion and virulence phenotypes of RGVC isolates.

Isolate	Hcp Pellet	Hcp Supernatant	Eukaryotic Killing	Prokaryotic Killing
V52	+++	+++	+++	+++
DL2111	−	−	−	−
DL2112	−	−	−	−
DL4211	+++	+++	++	+++
DL4215	+++	+++	+++	+++

## Results

### RGVC Isolates Exhibit T6SS-Mediated Antimicrobial Properties

We previously demonstrated that clinical *V. cholerae* O37 serogroup strain V52 uses its T6SS to kill *E. coli* and *Salmonella* Typhimurium [6]. To determine the role of the T6SS in environmental strains, we employed two different types of *V. cholerae* isolated from the Rio Grande: smooth isolates with distinct O-antigens as part of their lipopolysaccharides (LPS), and rough isolates that lack O-antigen (Table 3). Due to concerns that rough bacteria are genetically unstable because the lack of O-antigen allows the uptake of chromosomal DNA [24], we assessed the virulence potential of two separately isolated but genetically identical rough isolates DL2111 and DL2112 (as determined by deep sequencing (Illumina platform) of a polymorphic 22-kb fragment [Genbank accession numbers JX669612 and JX669613]) to minimize the chance of phenotypic variation due to genetic exchange.

To determine whether environmental RGVC *V. cholerae* are capable of killing bacteria, we performed an *E. coli* killing assay (Figure 1). RGVC isolates and *E. coli* strain MG1655 were spotted on LB nutrient agar plates, and the number of surviving MG1655 cells was determined after a 4-hour incubation at 37°C. V52 and V52Δ*vasK* were used as virulent and avirulent controls, respectively. The presence of V52 resulted in an average ∼5-log reduction of viable *E. coli*. Smooth isolates DL4211 and DL4215 killed *E. coli* at levels comparable to V52 (Figure 1). In contrast, both rough isolates, DL2111 and DL2112, were unable to kill *E. coli* prey. In summary, smooth RGVC isolates readily killed *E. coli* while rough RGVC isolates appeared to be attenuated.

### RGVC Isolates Display T6SS-Mediated Virulence Towards *D. discoideum*

The clinical *V. cholerae* O37 serogroup strain V52 displays T6SS-dependent cytotoxicity towards the social amoeba *D. discoideum* [4]. We tested whether RGVC isolates were also capable of evading amoeboid grazing by killing the eukaryotic predator. RGVC isolates were plated together with amoebae on nutrient agar plates that exclusively support bacterial growth. For amoebae to survive on agar plates, they must obtain nutrients from phagocytosed bacteria. This amoeboid grazing behavior on bacteria results in the formation of plaques–clear zones in the bacterial lawn that are devoid of bacteria [25]. The T6SS mediates bacterial virulence towards *D. discoideum* and abrogates plaque formation. Wild-type V52 and *Klebsiella pneumoniae* were used as virulent (no plaques) and avirulent (plaque formation) controls, respectively. Smooth isolates DL4211 and DL4215 killed *D. discoideum* at levels comparable to V52. In contrast, rough DL2111 and DL2112 did not kill *D. discoideum* similar to the T6SS-null mutant V52Δ*vasK* and the avirulent *Klebsiella pneumoniae* negative control (Figure 2).

### Expression of Hcp in RGVC Isolates

Next, we set out to test whether RGVC isolates were able to produce and secrete the T6SS hallmark protein Hcp because experimental results presented thus far suggested that *V. cholerae*'s ability to kill bacterial competitors or eukaryotic predators [6] could be mediated by the T6SS. As shown in Figure 3, smooth isolates DL4211 and DL4215 produced Hcp at sufficient levels to be detected by western blots probed with Hcp antiserum. In contrast, rough isolates did not produce or secrete Hcp. The presence of Hcp correlated with virulence as the smooth isolates secreted Hcp (Figure 3) and killed *E. coli* (Figure 1) as well as *D. discoideum* (Figure 2), while rough isolates did not produce Hcp and appeared to be attenuated.

### RGVC Isolates Engage in T6SS-Mediated Secretion and Virulence

To determine whether killing of *E. coli* (Figure 1) and *D. discoideum* (Figure 2) depends on a functional T6SS, we performed killing assays and plaque assays with DL4211Δ*vasK* and DL4215Δ*vasK* as a predator. VasK is an inner membrane protein believed to provide the energy for T6SS-mediated secretion [26], [27]. VasK is, therefore, crucial for a functional T6SS. As shown in figure 4A, parental V52, DL4211, and DL4215 constitutively produced and secreted Hcp, while deletion of *vasK* blocked secretion but not synthesis of Hcp. To complement the *vasK* chromosomal deletion, *vasK* from V52 was cloned downstream of an arabinose-inducible promoter in the plasmid pBAD24 and introduced into DL4211Δ*vasK* (DL4211Δ*vasK*/pvasK) and DL4215Δ*vasK* (DL4215Δ*vasK*/pvasK). *Trans* complementation of *vasK* restored Hcp secretion in V52 and the two smooth isolates (Figure 4A). To assess the role of T6SS in killing *E. coli*, we incubated *E. coli* with various *V. cholerae* isolates and determined the number of surviving *E. coli* after a 4-hour incubation at 37°C (Figure 4B). *VasK* mutants of V52, DL4211, and DL4215 lost their ability to kill *E. coli*, but providing *vasK in trans* restored virulence. Furthermore, amoebae were unable to form plaques in lawns of V52, DL4211, and DL4215, but did so in lawns of V52Δ*vasK*, DL4211Δ*vasK* and DL4215Δ*vasK* (Figure 4C). Complemented isolates, V52Δ*vasK*/pvasK, DL4211Δ*vasK*/pvasK and DL4215Δ*vasK*/pvasK, regained virulence towards *D. discoideum* (Figure 4C). Although the wild-type phenotype of DL4211 could not be fully complemented by episomal expression of *vasK*, the complemented phenotype is statistically significant (unpaired t-test, p = 0.0116). We conclude that smooth RGVC isolates conferred T6SS-mediated virulence towards *E. coli* and *D. discoideum*, demonstrating that the virulence phenotype described in Figures 1 and 2 is T6SS-dependent.

### Rough RGVC Isolates Carry Unique vasH Sequences

We previously showed that the global transcriptional activator VasH is essential for expression of *hcp* and other T6SS genes. As the rough isolates failed to synthesize Hcp (Figure 3), we tested whether these isolates carried a nonfunctional *vasH* allele. The 1594 nucleotide-long *vasH* sequences of V52 and RGVC isolates were PCR-amplified and their polypeptide sequences aligned. The rough RGVC isolates were missing a guanine in codon 157 (ΔG157) which resulted in a frameshift. To include *vasH* of the rough isolates in our comparative analysis, we restored the *vasH* reading frame by *in-silico* insertion of G157. We found that all RGVC VasH sequences aligned with V52 and N16961 as well as with each other (Figure 5). Therefore, *vasH* is conserved in environmental (RGVC), pandemic (N16961), and endemic (V52) *V. cholerae* strains. The repaired *vasH* open reading frame closely resembled *vasH* from N16961 with only two unique substitutions (Q278L and T456I). Smooth RGVC isolate DL4211 carried an intact VasH gene identical to N16961; DL4215 differed from N16961 and V52 by three and four residues, respectively (Table 3). Substitutions of histidine to aspartic acid at position 116 (H116D) and threonine to alanine at position 449 (T449A) appear to be common substitutions that are also present in N16961 (Figure 5). In conclusion, RGVC isolates carry a VasH gene related to the El Tor version with the characteristic D116 and A449 residues (Figure 5). However, rough *V. cholerae* isolates carried a nonsense mutation and are likely to produce a truncated 63 amino acid-long VasH mutant protein.

### VasH Complementation in Rough RGVC Isolates

We tested whether heterologous expression of *vasH* in the T6SS-silent RGVC isolates DL2111 and DL2112 restored T6SS-dependent protein synthesis/secretion. Myc-tagged *vasH* from V52 was cloned into pBAD18 to episomally express *vasH*. V52Δ*vasH*/pBAD18-vasH::myc was used as a control for the arabinose-dependent expression of *vasH*. As shown in Figure 6, episomal *vasH*::myc expression in V52Δ*vasH* induced Hcp production and subsequent secretion, while only synthesis but not secretion was restored in the rough RGVC isolates.

### Smooth RGVC Isolates Use Their T6SS to Compete with Natural Neighbors

Because RGVC isolates with active T6SSs kill *E. coli*, we hypothesized that RGVC isolates use their T6SS to compete with other bacteria in their environmental niche. To test this hypothesis, we isolated three environmental bacterial non-*V. cholerae* strains from estuaries where the Rio Grande meets the Gulf of Mexico. Sequencing of 16S-rRNA identified these bacterial species as *Vibrio communis*, *Vibrio harveyi*, and *Pseudoalteromonas phenolica* (data not shown). We then tested whether DL4211 and DL4215 were able to kill these environmental bacteria in a T6SS-dependent fashion. As shown in Figure 7, both DL4211 and DL4215 killed all three environmental isolates. The observed killing required a functional T6SS, as isogenic *vasK* mutants lost their ability to kill. Killing of the environmental bacteria was restored by complementing the *vasK* mutant backgrounds with episomal *vasK in trans*. Therefore, we propose that constitutive expression of T6SS genes provides smooth RGVC isolates with the means to kill both their bacterial neighbors and potential eukaryotic predators.

### Smooth RGVC Isolates Use Their T6SS for Intraspecific Competition

*V. cholerae* O37 strain V52 kills *E. coli* and *S*. Typhimurium, but is unable to kill other *V. cholerae*, including the O1 serogroup N16961 (El Tor) and O395 (classical biotype) strains [6]. Accordingly, the T6SS^+^ isolates V52, DL4211 and DL4215 also exhibited immunity, because we did not observe a decline in viable CFUs when we recovered these isolates from single-isolate spots on LB agar plates after a 4-hour incubation (data not shown). We hypothesized that *V. cholerae* employs an immunity system that provides protection against T6SS-mediated toxicity. A functional link between T6SS and toxin/antitoxin systems has been established in *Pseudomonas aeruginosa* and *Burkholderia* species [28], [29], which employ antitoxin proteins to counteract T6SS effectors [28]. VCA0124, an open reading frame downstream of the T6SS effector gene *vgrG3* (VCA0123), has been implicated as an antitoxin gene in *V. cholerae* [30]. As RGVCs killed close relatives such as *V. harveyi* (Figure 7), we wondered if the RGVC isolates have the ability to kill each other. We hypothesized that if RGVC isolates use different toxins (and antitoxins), the T6SS might be used for intraspecific competition. We predicted that immunity of an RGVC isolate would be lost when approached by a *V. cholerae* bacterium with a different set of T6SS toxins to which the former lacks the corresponding antitoxin gene. To test this hypothesis, we mixed V52, DL4211, and DL4215 (predators) with smooth and rough RGVC isolates as prey bacteria. To eliminate the killing activity of smooth T6SS^+^ prey, we used *vasK*-deficient mutants with a disabled T6SS as prey. Rough wild-type RGVC isolates were used as prey since they do not express Hcp (Figure 3) and are thus T6SS-negative. Following a 4-hour coincubation, we determined the number of surviving prey. T6SS-negative prey bacteria were not killed by their isogenic T6SS^+^ parent strain, but were killed by other T6SS^+^ isolates (Figure 8A–C). Exposure to a predator with a disabled T6SS resulted in about 10^8^ surviving prey bacteria. Similar numbers of surviving prey were obtained when the prey was mixed with an isogenic strain that was marked with a different antibiotic resistance cassette (data not shown). Thus, killing of T6SS-negative prey required a functional T6SS. Surprisingly, the *vasK* mutant of DL4215 displayed virulence towards V52Δ*vasK*, but not against DL4211Δ*vasK* or a differently-marked DL4215Δ*vasK* sister strain (Figure 8C). Since DL4215Δ*vasK* does not kill *V. communis*, *V. harveyi*, or *P. phenolica* (Figure 7), we hypothesize that DL4215 exhibits some degree of selective T6SS-independent antimicrobial activity against V52Δ*vasK*.

In conclusion, *V. cholerae* uses its T6SS not solely for competition with bacterial neighbors (Figure 7), but also for competition within its own species (Figure 8D).

## Discussion

We examined environmental smooth and rough *V. cholerae* isolates (RGVCs) collected at two locations along the Rio Grande to study T6SS regulation in *V. cholerae* exposed to microbial competitors and predators.

Our study showed that smooth RGVC isolates use their T6SS to kill other Gram-negative bacteria isolated from the Rio Grande delta. Deletion of the T6SS gene *vasK* resulted in a loss of bacterial killing. Importantly, the killing phenotype was restored by *vasK* complementation *in trans*. The requirement of VasK for killing implies that a constitutively active T6SS provides smooth RGVC isolates with a competitive advantage compared to their bacterial neighbors. By killing other bacteria, RGVC isolates might enhance their own survival in their environmental niche. In addition, we found that *V. cholerae* isolates use their T6SS to compete against each other.

In our experiments, Hcp synthesis and secretion correlated with eukaryotic and prokaryotic host cell killing (Table 4). For example, smooth Hcp-secreting RGVC isolates DL4211 and DL4215 (Figure 3) displayed full virulence towards *E. coli* (Figure 1) and *D. discoideum* (Figure 2). Rough RGVC isolates with their frameshift mutations in the T6SS transcriptional activator gene *vasH* did not produce or secrete Hcp, and their virulence was attenuated. Sequencing and gene alignments of the T6SS transcriptional activator *vasH* in rough strains indicated a missing guanine at position 157 in rough isolates, resulting in a frameshift mutation. Because VasH was recently implicated in regulating both the large and auxiliary T6SS gene clusters in *V. cholerae* O395 [20], we speculated that the *vasH* frameshift mutation in the rough isolates silences T6SS expression. However, *trans*-complementation of the *vasH* mutation by episomal expression of V52′s *vasH* restored synthesis but not secretion of the T6SS hallmark protein Hcp (Figure 6). *Trans*-complementation with *vasH* from N16961, which is more closely related to *vasH* from RGVC isolates DL2111 and DL2112 (Figure 5), restores Hcp synthesis and secretion in a *vasH* mutant of V52, but only restores Hcp synthesis (and not secretion) in a *vasH* mutant of N16961 [19]. Thus, we believe that the inability to restore Hcp secretion in rough strains is not a reflection of the polymorphic nature of VasH.

At this time, it is unclear whether selective pressures for T6SS regulation exist that drive constitutive T6SS expression in smooth isolates and disable T6SSs in rough *V. cholerae* strains. *V. cholerae* LPS's O-antigen has been shown to induce protective immune responses in humans and experimental animals [31]–[35]. To counteract the host immune response, *V. cholerae* may use its T6SS to kill phagocytic immune cells such as macrophages [9]. Because rough isolates lacking O-antigen are frequently isolated from convalescent cholera patients [36], repression of O-antigen biosynthesis may represent an immune evasion mechanism for *V. cholerae* [37]. Such evasion would allow the pathogen to persist in the host, perhaps in a subclinical state as rough *V. cholerae* have been shown to be avirulent. In this scenario, rough *V. cholerae* does not require a functional T6SS, but tolerates mutations that disable its expression. Rough isolates have been shown to revert to a smooth, virulent state [37] but it remains to be determined whether newly reverted smooth bacteria restore expression of their disabled T6SSs. We did not observe restoration of the T6SS in rough isolates through uptake and homologous recombination of chromosomal DNA from a T6SS^+^ donor, because rough isolates remained T6SS-negative in the presence of smooth T6SS^+^ *V. cholerae* strain V52 (data not shown).

El Tor strains possess a tightly controlled T6SS [17] and thus differ from the smooth RGVCs that express the T6SS constitutively. As pandemic strains are believed to originate from environmental strains, we speculate that constitutive T6SS expression is prevalent in *V. cholerae* exposed to microbial competitors and predators until virulence factors such as cholera toxin and toxin-coregulated pilus genes are acquired. However, how pandemic *V. cholerae* regulate expression of T6SS during their complex life cycle remains to be determined.

It is becoming increasingly clear from our investigation and other reports [6], [28]–[30], [38] that T6SS-expressing *V. cholerae* deploy bactericidal effector proteins. Therefore, T6SS expression is likely tied to a protective mechanism, a form of T6SS-immunity that prevents the effector proteins from harming bacteria within a clonal population. We postulate that V52, DL4211, and DL4215 employ unique sets of toxin/antitoxin gene products and therefore form distinct compatibility groups. Members of a T6SS compatibility group could coexist because they encode antitoxins that match the cognate toxins. Conversely, members of different T6SS compatibility groups kill each other since the antitoxins of one compatibility group do not protect against the toxins of the other group. Hence, T6SS-mediated selective interstrain killing allows *V. cholerae* to distinguish self from nonself. This form of kin selection may permit the evolution of distinct lineages, including those that give rise to toxigenic strains. The observations presented in this study indicate that the T6SS contributes to *V. cholerae*'s pathogenesis and fitness by providing an advantage in interspecific competition with eukaryotes or prokaryotes, and intraspecific competition with *V. cholerae* strains.
